# Improving treatment efficiency via photon optimizer (PO) MLC algorithm for synchronous single‐isocenter/multiple‐lesions VMAT lung SBRT

**DOI:** 10.1002/acm2.12721

**Published:** 2019-09-20

**Authors:** Lana Sanford, Damodar Pokhrel

**Affiliations:** ^1^ Medical Physics Graduate Program University of Kentucky Lexington KY USA; ^2^ Department of Radiation Medicine University of Kentucky Lexington KY USA

**Keywords:** lung SBRT, MLC, photon optimizer, single‐isocenter/multi‐lesions, VMAT

## Abstract

**Purpose:**

Elderly patients with multiple primary or oligometastases (<5 lesions) lesions with associated co‐morbidities may not retain their treatment position for the traditional long SBRT treatment time with individual isocenters for each lesion. Treating multiple lesions synchronously using a single‐isocenter volumetric arc therapy (VMAT) plan would be more efficient with the use of the most recently adopted photon optimizer (PO) MLC algorithm and improve the patient comfort. Herein, we quantified the clinical performance of PO versus its predecessor progressive resolution optimizer (PRO) algorithm for single‐isocenter/multiple‐lesions VMAT lung SBRT.

**Materials and methods:**

Fourteen patients with metastatic non‐small‐cell lung cancer lesions (two to five, both uni‐ and bilateral lungs) received a highly conformal single‐isocenter co/non‐coplanar VMAT (2–6 arcs) SBRT treatment plan. Patients were treated with a 6X‐FFF beam and Acuros algorithm with a single‐isocenter placed between/among the lesions, using PO for MLC optimization. Average isocenter to tumor distance was 5.5 ± 1.9 cm. Mean combined PTV derived from 4D‐CT scans was 38.7 ± 22.7 cc. Doses were 54 Gy/50 Gy in 3/5 fractions prescribed to 70%–80% isodose line so that at least 95% of the PTV receives 100% of prescribed dose. Plans were re‐optimized using PRO algorithm. Plans were compared via ROTG‐0915 protocol criteria for target conformity, heterogeneity and gradient indices, and dose to organs‐at‐risk (OAR). Additionally, total number of monitor units (MU), modulation factor (MF) and beam‐on time were compared.

**Results:**

All plans met SBRT protocol requirements for target coverage and OAR doses. Comparison of target coverage and dose to the OAR showed no statistical significance between the two plans. PO had 1042 ± 753 (*P* < 0.001) less MU than PRO resulting in a beam‐on time of about 0.75 ± 0.5 min (*P* < 0.001) less, on average. For similar dose distribution, a significant reduction of beam delivery complexity was observed with PO (average MF = 3.7 ± 0.7) vs PRO MLC algorithm (average MF = 4.4 ± 1.3) (*P* < 0.001).

**Conclusions:**

PO MLC algorithm improved treatment efficiency without compromising plan quality when compared to PRO algorithm for single‐isocenter/multi‐lesions VMAT lung SBRT. Shorter beam‐on time can potentially reduce intrafraction motion errors and improve patient compliance. PO MLC algorithm is recommended for future clinical lung SBRT plan optimization.

## 1 INTRODUCTION

With recent technological advances, SBRT treatment to solitary primary or metastatic lung lesions for medically inoperable non‐small‐cell lung cancer (NSCLC) patients is safe, effective and has a high cure rate comparable to surgery.^1^, ^2^, ^3^, ^4^ SBRT can be beneficial for elderly patients.^5^ However, elderly patients who developed multiple primary or oligometastases (<5 lesions) lung lesions with associated co‐morbidities may not retain their treatment position for traditional long SBRT treatment times with an individual isocenter placed for each lesion. Treating multiple lung lesions synchronously with a single‐isocenter plan, either using intensity‐modulated radiation therapy (IMRT) or volumetric arc therapy (VMAT), has been studied by a few researchers.^6^, ^7^, ^8^, ^9^ Furthermore, utilizing flattening filter free (FFF) beam^10^ for single‐isocenter multiple‐lesion VMAT lung stereotactic body radiation therapy (SBRT) treatment was fast and efficient, improved the patient comfort and is gaining popularity in clinical practice.^11^, ^12^, ^13^

Recently, Varian Eclipse treatment planning system (TPS, Varian Medical Systems, Palo Alto CA, Version 13.6) has implemented a new multi leaf collimators (MLC) optimization algorithm, called photon optimizer (PO). Photon optimizer was created to be more efficient for IMRT/VMAT optimization over its predecessor, progressive resolution optimizer (PRO).^14^ The main difference between PO and PRO algorithms is that PO uses a new structure model. For PO, the structures, dose‐volume histogram calculations and dose sampling are defined spatially using a single matrix over the image instead of a point‐cloud model defining structures that was used in the PRO algorithm. In this configuration, PO algorithm under‐samples voxels at the periphery of the target. However, PO configuration uses multiresolution dose calculation approach to increase the dose calculation accuracy. Fixed voxel resolutions of 1.25, 2.5 or 5 mm can be used during multiresolution optimization. For a single‐lesion treatment, a few investigators have reported dosimetric differences between PO and PRO optimization for IMRT/VMAT plans.^15^, ^16^, ^17^, ^18^ For instance, the advantages and limitations of PO algorithm compared to its predecessor PRO for IMRT plans were evaluated by Binny et al.^16^ Eleven plans including prostate, brain, and head and neck treatments were optimized using both PO and PRO algorithms. For similar target coverage and dose to critical structures, they reported that PO algorithm gave higher MLC variability and more monitor units. However, Liu et al.^18^ compared PO with PRO algorithms for VMAT planning of lung SBRT and brain stereotactic treatments. Their retrospective study included 20 lung SBRT patients (10 received 54 Gy in 3 fractions and 10 received 50 Gy in 5 fractions) and 10 brain stereotactic patients received 25 Gy in five fractions. For identical target coverage, PO algorithm provided comparable plan quality to PRO, with less MLC complexity, thus improving the treatment delivery and contradicting Binny et al.^16^

Although dosimetric differences with PO algorithm for a single‐lesion treatment with SBRT have been studied previously by Liu et al.^18^, the dosimetric impact and treatment delivery complexity of this algorithm with a FFF‐beam in the treatment of multiple lesions simultaneously using a single‐isocenter VMAT lung SBRT plan has not yet been reported. When using a single‐isocenter for VMAT lung SBRT, the MLCs must travel a longer distance to provide adequate coverage to each lesion simultaneously. Moreover, due to under sampling of the voxels at the periphery of each tumor by the PO algorithm, this distance could cause higher nontarget normal tissue dose to the organs‐at‐risk (OAR) adjacent to the tumor. This prompted us to quantify the effect of PO MLC algorithm for our clinical implementation of single‐isocenter/multi‐lesions VMAT lung SBRT approach. Dose to radiosensitive nontarget OAR is a major concern in VMAT lung SBRT treatment,^19^, ^20^ specifically while treating multifocal lesions synchronously. The goals of this technical report were to quantify the dosimetric performance of PO algorithm for FFF‐beam in the SBRT treatment of multifocal lung lesions using a single‐isocenter plan and to investigate the further improvements of the delivery efficiency with MLC movements. We have retrospectively evaluated 14 single‐isocenter/multifocal (2–5 lesions) NSCLC patient's plans who underwent VMAT‐SBRT treatment in our clinic using the PO algorithm. For comparison, the clinical PO‐VMAT plans were re‐optimized with the PRO algorithm with identical beam geometry, planning objectives and optimization parameters. The original PO‐VMAT and re‐optimized PRO‐VMAT plans were compared by lung SBRT protocol compliance, target conformity, gradient indices and dose to OAR per RTOG guidelines.^3^

## 2 MATERIALS AND METHODS

### 2.A Patient population and treatment planning

This retrospective study included 14 patients with metastatic non‐small‐cell lung lesions. Each patient had 2–5 synchronous lung lesions. The patients were immobilized using Body Pro‐Lok^TM^ platform (CIVCO system, Orange City, IA) in the supine position, arms above their head with abdominal compression. All planning computed tomography (CT) images were acquired on a GE Lightspeed 16 slice CT scanner (General Electric Medical Systems, Waukesha, WI). CT images were acquired with 512 × 512 pixels at 2.5 mm slice thickness. All patients underwent a free breathing scan followed by a 10 phase four dimensional‐CT scan using Varian's Real Time Position Management Respiratory Gating System (version 1.7). Internal target volumes (ITVs) were delineated on the three dimensional CT images with reference to the maximum intensity projection (MIP) images and the planning target volumes (PTVs) were created by adding a 5 mm uniform margin around the corresponding ITV. Mean combined PTV derived from four‐dimensional computed tomography (4D‐CT) scan was 38.7 ± 22.7 cc. The critical structures, such as bilateral lungs excluding the ITV (normal lung), spinal cord, ribs, heart, trachea and bronchus, esophagus, and skin were delineated on the free‐breathing CT images.

A single‐isocenter was placed approximately between/among the tumors in each patient. Average isocenter to tumors distance was 5.6 ± 1.9 cm. Highly conformal, clinically optimal VMAT treatment plans were generated on the free‐breathing CT scan using 2–6 co/non‐coplanar full/partial arcs (5°–10°, couch kicks were used for non‐coplanar partial arcs) for the Truebeam linear accelerator (Varian, Palo Alto, CA) with millennium MLC and a 6MV‐FFF (1400MU/min) beam. All clinical plans were optimized in Eclipse (version 13.6) with PO algorithm using a fixed 2.5 mm voxel resolution. The standard millennium 120 leaves with 5 mm leaf width were used for treatment planning and delivery. For 6X‐FFF beam, MLC transmission and leakage modeled in Eclipse was 1.5% in addition to 1.1 mm dosimetric leaf gap. PO sparsely samples a point dose cloud model for defining structures and spatial dose using one single matrix over the image. For each arc, collimator angles were chosen such that the opening of the MLC between/among tumors was minimized for each patient. Additionally, the jaw tracking option was chosen during VMAT plan optimization to further minimize the non‐target dose. Advanced Acuros‐based dose calculation and dose to medium was used. A dose of 54 or 50 Gy in 3 and 5 fractions was prescribed to 70%–80% isodose line such that at least 95% of the each PTV received the prescription dose. In addition to optimization ring structures, the generalized normal tissue objective (NTO) parameters were used to control the gradients for each target. Planning objectives for the OAR were per RTOG 0915 guidelines.^3^ The main tumor characteristics of the patients included in this study is shown in Table 1.

**Table 1:** Main tumor characteristics of the patients included in this study.

Parameters	Mean ± SD (range or no. of patients)
Combined PTV (cc)	38.7 ± 22.7 (15.9–91.8)
Prescription dose (each lesion)	54 Gy in 3 fractions (7 patients)
	50 Gy in 5 fractions (7 patients)
Normal lung volume (cc)	3881 ± 1161 (1893–6543)
Isocenter to tumors distance (cm)	5.5 ± 1.9 (3.4–9.5)
Laterality (left/right/bilateral lung)	(5/3/6 patients)

### 2.B Quality assurance and treatment delivery

Planning and delivery dose agreement for the PO plans was assessed using an Octavius phantom (PTW, Freiburg, Germany). For Octavius quality assurance (QA) plans, the average pass rates for the single‐isocenter/multiple‐lesion VMAT lung SBRT plans were 98.8 ± 2.5% for 3%/2 mm clinical gamma pass rate criteria with a maximum point dose measurement was 1.0 ± 0.7%. The beam‐on time (BOT) was calculated using the average delivered dose rate of 1400 MU/min for these plans. The delivered dose rate was confirmed by reviewing each VMAT arc for all patients under the MLC properties in Eclipse. Furthermore, average delivered dose rate of 1400 MU/min was visually observed (for each arc) during VMAT‐QA delivery at Truebeam Linac for all single isocenter/multiple‐lesion lung SBRT plans.

Before delivering each PO VMAT lung SBRT treatment, a daily QA check on kilovoltage to megavoltage imaging isocenter coincidence was performed, including IsoCalc measurement for precise and accurate target localization. Our IsoCalc localization accuracy for Truebeam was <0.5 mm. All the QA procedures were in compliance for SBRT treatment delivery. The patients were set up using daily cone beam CT scan following an image‐guidance SBRT procedure established in our clinic. Patients were treated every other day following in‐house lung SBRT protocol.

### 2.C PRO VMAT plan

For comparison, the clinical PO VMAT treatment plans for all SBRT patients were retrospectively re‐optimized using a PRO MLC algorithm. Identical beam geometry, dose calculation algorithm and planning objectives were used in the PRO and PO plan including the NTO parameters and ring structures. The PRO plan received the same target coverage as the clinical PO plan. Additionally, other treatment plan optimization parameters such as maximum number of iterations, convergence mode, dose reporting mode, and calculation grid‐size (etc.) were kept identical between the two plans.

### 2.D Plan analysis

The dose‐volume histograms (DVHs) and isodose curves of PO and PRO plans were compared. The conformity index (CI), heterogeneity index (HI), gradient index (GI), gradient distance (GD), and D_2cm_ were calculated per RTOG 0915 requirement. The dose to the normal lung was evaluated using V5 Gy, V10 Gy, V20 Gy, mean lung dose (MLD), and maximum dose to 1000 cc of lungs. Furthermore, dosimetric disparities were evaluated for spinal cord, heart, trachea and bronchial tree, esophagus, ribs and skin following RTOG guidelines. Total number of monitor units (MU), modulation factor (MF), and measured BOT were compared. The MF is defined as the total number of MU divided by the prescription dose in cGy. BOT was calculated using total MU divided by the average delivered dose‐rate. Statistical analysis was performed using Microsoft Excel (Microsoft Corp., Redmond, WA) data analysis program. Paired sample t‐test was used to evaluate parameters for PO versus PRO plans using *P* < 0.05 (two‐sided).

## 3 RESULTS

Plans were normalized to receive the same target coverage (i.e., PTV D95 = 100%). The dose distribution in the target volumes remained similar with no significant differences in conformity between PO and PRO plans. Table 2 shows the compiled data. Both plans met SBRT protocol requirement for target coverage and OAR doses. Statistically insignificant differences were observed for doses to lung parameters (V5 Gy, V10 Gy, V20 Gy, MLD, and maximum dose to 1000 cc of lung) with PO compared to PRO plan.

**Table 2:** Analysis of the dosimetric and delivery parameters for all 14 lung SBRT patients treated with a single‐isocenter/multiple‐lesions volumetric arc therapy (VMAT) plan. Mean ± SD (range) and *P*‐values were reported for photon optimizer (PO) and progressive resolution optimizer (PRO) plans. n. s. = not significant. Significant values are highlighted in bold. SD = standard deviation.

Parameter	PO algorithm	PRO algorithm	*P*‐value
Combined PTV and V20 Gy			
CI	1.04 ± 0.05 (1.00–1.16)	1.03 ± 0.12 (1.00–1.13)	*n. s.*
HI	1.23 ± 0.03 (1.20–1.31)	1.22 ± 0.04 (1.14–1.29)	*n. s.*
GI	5.5 ± 1.4 (4.1–6.4)	5.4 ± 1.2 (4.0–6.0)	*n. s.*
D2cm (%)	52.5 ± 6.0 (43–67)	53.0 ± 7.6 (41–68)	*n. s.*
GD (cm)		1.48 ± 0.25 (1.22–2.03)	*n. s.*
V20Gy (%)	6.4 ± 3.1 (2.5–13.5)	6.5 ± 3.3 (2.42–13.7)	*n. s.*
Max dose to OAR (Gy)			
Skin	17.03 ± 3.7 (11.0–21.0)	17.26 ± 3.1 (11.9–21.7)	*n. s.*
Ribs	41.1 ± 12.3 (25.1–59.0)	39.84 ± 12.5 (23.2–58.9)	*n. s.*
Spinal cord	10.45 ± 3.6 (5.8–15.5)	10.53 ± 4.2 (4.4–16.0)	*n. s.*
Heart	23.65 ± 11.3 (7.9–52.0)	23.03 ± 11.4 (8.8–51.5)	*n. s.*
Bronchus	23.35 ± 13.2 (4.7–50.2)	23.13 ± 13.5 (5.7–51.8)	*n. s.*
Trachea	8.91 ± 6.7 (0.5–19.2)	9.93 ± 7.9 (0.5–20.0)	*n. s.*
Esophagus	17.75 ± 7.5 (8.4–33.0)	18.46 ± 7.7 (9.8–34.3)	*n. s.*
Delivery parameter			
Total MU	5161 ± 2257 (2784–10727)	6203 ± 2869 (3437–13012)	**0.0005**
MF	3.66 ± 0.9 (2.8–5.9)	4.41 ± 1.26 (3.1–7.3)	**0.0001**
BOT (min)	3.69 ± 1.61 (2.0–7.7)	4.43 ± 2.05 (2.5–9.3)	**0.0005**

**Figure 1:**
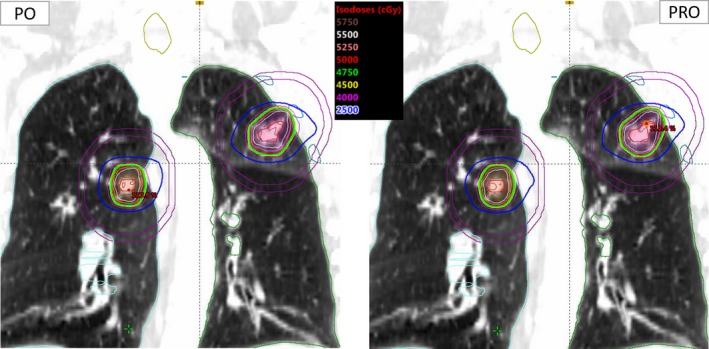
The isodose distribution is shown for photon optimizer (PO) (left) and progressive resolution optimizer (PRO) (right) for an example case patient who was treated for bilateral lung lesions, synchronously using 2‐full co‐planner arcs. This patient received a synchronous SBRT treatment to a total dose of 50 Gy to each lesion in 5 fractions. The single‐isocenter location is shown by the cross‐hair. Tumors were located in bilateral lungs. Isocenter to tumor distance was an average of 5.8 cm. Combined planning target volume (PTV) was 24.5 cc with lesion 1, PTV1 (left lung) = 18.0 cc and lesion 2, PTV2 (right lung) = 6.5 cc. PO and PRO algorithms provided similar SBRT dose distributions to each lung lesion.

Also, dose to 0.35 cc of spinal cord, 15 cc of heart, 5 cc of esophagus, 4 cc of bronchial tree and trachea, 1 cc of ribs and dose to 10 cc of skin met SBRT protocol guidelines with both plans and were statistically insignificant (not shown in Table 2). However, the total number of MU, MF, and BOT show statistically significant differences between the two plans (see Table 2). PO algorithm provided 1042 ± 753 (*P* < 0.001) less MU than PRO, resulting in a BOT of about 0.7 ± 0.5 min (*P* < 0.001) less, on average. For a similar dose distribution, significant reduction of beam delivery complexity was observed with PO algorithm (average MF = 3.7 ± 0.7) versus PRO (average MF = 4.4 ± 1.3) with *P* < 0.001. An example isodose distribution in the coronal view is shown in Fig. 1.

**Figure 2:**
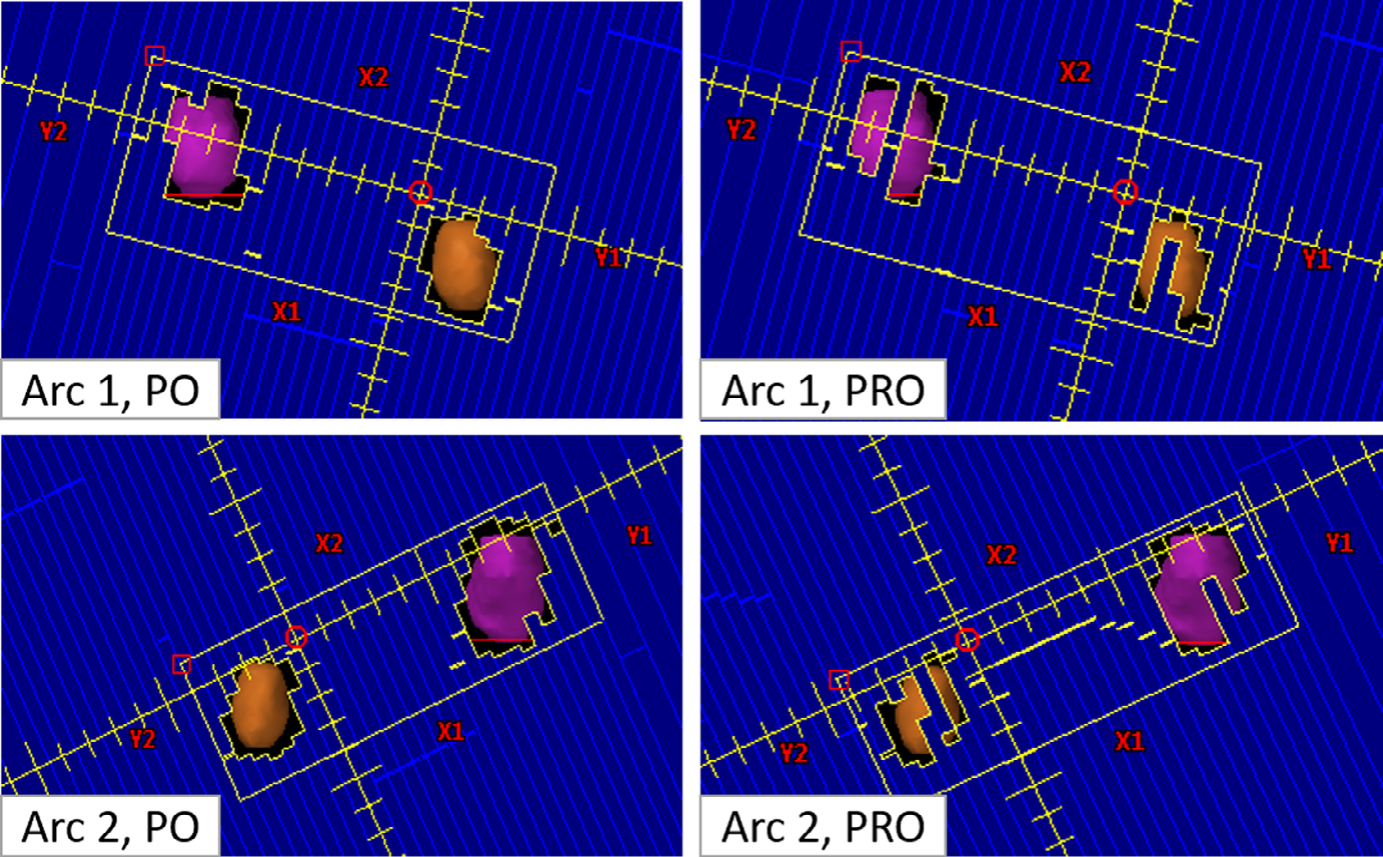
Comparisons of the selective corresponding MLC control points (one control point each from arc 1 and arc 2) between the photon optimizer (PO) and progressive resolution optimizer (PRO) algorithms for the same patient plan (this patient was treated using 2‐full co‐planner arcs) shown in Fig. 1. PO multi leaf collimators (MLC) algorithm (left panel) and PRO MLC algorithm (right panel) were shown. Although both MLC optimizers provided similar target coverage and organs‐at‐risk doses, PO delivers treatment relatively faster and potentially more accurately due to the less MLC modulation. PO control points showing larger MLC opening at the PTV(s) margin, compared to the corresponding PRO control points, was associated with relatively smaller monitor units, modulation factor and shorter beam‐on time.

An example of the corresponding MLC control points for PO and PRO algorithms of a representative patient is shown in Fig. 2. It has been observed that PO algorithm reduced the small MLC opening significantly when compared with PRO algorithm, which was indicated by the decrease in the MF values with PO. Eliminating smaller MLC openings with VMAT plans could potentially lead to more accurate dose delivery due to the reduction in small‐field dosimetric uncertainty in the beam model. Larger MLC opening and less total MU led to shorter beam on time while using PO algorithm that was desirable for multiple lung lesions SBRT treatment.

**Figure 3:**
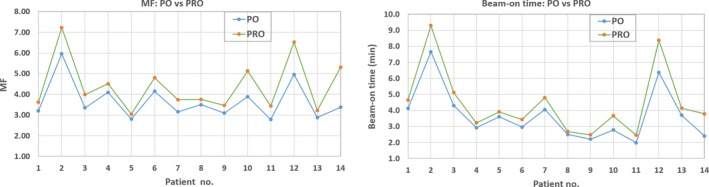
Left panel: modulation factor (MF) for photon optimizer (PO) vs progressive resolution optimizer (PRO) algorithms on a per‐patient basis for all 14 patients treated with single‐isocenter/multiple‐lesion volumetric arc therapy lung SBRT plan. Mean values of MF for PO and PRO algorithms were 3.66 ± 0.9 (ranged, 2.78–5.96) and 4.41 ± 1.26 (ranged, 3.05–7.23), respectively. Right panel: The corresponding beam‐on time for PO vs PRO. Mean values of beam‐on time for PO and PRO algorithms were 3.69 ± 1.61 min (ranged, 2.0–7.7 min) and 4.43 ± 2.05 min (ranged, 2.5–9.3 min), respectively, with PO algorithm significantly improving the beam‐on time.

The MF and the beam‐on time for PO vs PRO algorithms on per‐patient basis is shown in Fig. 3. For the given single‐isocenter/multi‐lesions lung SBRT plan, the total number of MU was reduced significantly while using PO algorithm for VMAT plan optimization, suggesting that the PO plan had smaller MF (*P* < 0.001). Because of this, the average beam‐on time for PO plans was 0.75 min less (maximum up to 2.0 min) than PRO plan due to less total MU.

**Figure 4:**
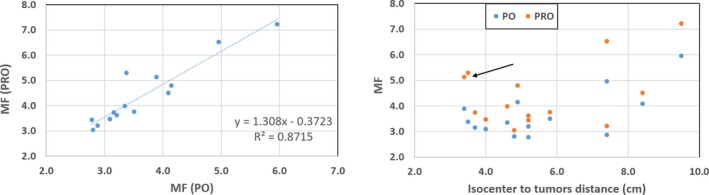
Scatter plots: progressive resolution optimizer (PRO)‐modulation factor (MF) as a function of photon optimizer (PO)‐MF (left panel) and MFs as a function of isocenter to tumors distance (right panel) for all 14 single‐isocenter/multi‐lesions VMAT lung SBRT patient plans. The PO‐MLC plans reduced the total number of MU significantly and hence increased the treatment delivery efficiency compared to its predecessor PRO algorithm. Furthermore, MF increases as a function of isocenter to tumors distance (see right panel), suggesting that the farther apart the tumors are, but for similar target(s) coverage, there is significantly less beam‐modulation with PO compared to PRO‐MLC algorithm.

The MF for PO vs PRO algorithms and the MF as a function of the isocenter to tumors distance is shown in Fig. 4. For the given lung SBRT plan, the total number of MU changed significantly while using PO‐MLC algorithm for plan optimization, suggesting that PO plans provided less beam modulation (see left panel) thus significantly affecting the beam‐on time. Furthermore, MF increases as a function of isocenter to tumors distance (see right panel in Fig. 4), suggesting that farther apart the tumors, PRO algorithm required significantly more MU to be delivered for the similar target coverage compared to OP algorithm. The black arrow in Fig. 4 (see right panel) shows that PRO's MF increases almost by a factor of 1.8 with 3‐ and 5‐lesions patient plans (we had one patient with 3‐lesion and one patient with 5‐lesions) even though the isocenter to tumors distance (average distance) was about 3.5 cm compared to the PO algorithm. However, more patient plans are needed to further validate PO algorithm to realize less beam modulation as function of number of lesions vs isocenter to tumors distance compared to PRO (as seen in our preliminary results). Therefore, it could provide further guidance about which case will benefit more from using PO algorithm, more lesions or fewer lesions, larger distance or shorter distance from the isocenter. To further validate PO algorithm, we are considering typical clinical cases that include a single‐isocenter VMAT stereotactic radiosurgery plan for multiple brain metastatic lesions.

## 4 DISCUSSION

In this technical report, we investigated the potential improvement of treatment delivery efficiency while utilizing PO algorithm in the treatment of single‐isocenter/multiple‐lesion VMAT lung SBRT patients. For similar target coverage, intermediate dose spillage and dose to the OAR, our PO VMAT plan provided lower number of MU, smaller MF and shorter beam‐on time compared to PRO plan (see Table 2). Most importantly, the beam‐on time was improved by 0.75 min, on average (maximum up to 2.0 min, see Fig. 3) with PO algorithm, compared to PRO algorithm. Our preliminary results also suggests that PO algorithm provided less beam‐modulation as a function of number of lesions and increasing distance to lesions compared to PRO algorithm in the single‐isocenter/multi‐lesions lung SBRT setting (see Fig. 4).

A few investigators have reported the dosimetric differences in PO algorithm for IMRT/VMAT planning in a digital phantom,^15^ conventional prostate, head and neck, and brain treatments,^16^ knowledge‐based planning to rectal cancer patients^17^ and a single‐lesion lung SBRT and stereotactic brain treatments.^18^ For instance, Jiang et al.^17^ evaluated the performance of PO over PRO for VMAT planning of 30 rectal cancer patients with or without knowledge‐based planning. A knowledge‐based model was generated using manually optimized PRO plans. They have shown that the overall reduction in OAR doses by 23.5% to 32.7% compared to the clinical plans suggesting that utilizing the RapidPlan model with PO algorithm (contributed by both RapidPlan model and PO algorithm) could be beneficial for future plan optimization. However, PO relative to PRO accounted for 1.5% to 3.8% OAR dose reduction suggesting that PO provided similar or better OAR dose without using RapidPlan model. Another study previously mentioned by Liu et al.^18^ compared the dosimetric impact of PO algorithm for single‐lesion lung SBRT and brain stereotactic treatment, and concluded that PO offers comparable dosimetry and reduced MLC complexity relative to the PRO algorithm.

While agreeing with aforementioned retrospective reports, our clinically optimized synchronous PO lung SBRT plans also exhibit similar target coverage and OAR sparing compared to PRO plans. Most importantly, PO algorithm provided smaller MF and shorter beam‐on time for the given complexity of single‐isocenter/multiple‐lesion VMAT lung SBRT plan. By optimizing a VMAT plan with PO algorithm, the smaller openings of the MLCs were eliminated leading to smaller total MU, MF, and consequently shorter beam on time. This suggests more accurate dose delivery due to the reduction in small‐field dosimetric uncertainty.^21^ Reducing the number of small MLC openings is important for improving delivery accuracy, especially with the MLC leaves traveling relatively longer distances between tumors in the synchronous treatments presented here. Even though PO under sampled voxels at the periphery of each tumor, the dosimetric differences were insignificant to the OAR with similar VMAT QA results and improvement of delivery efficiency.

In summary, the potential benefit of PO algorithm on Truebeam (with 6 MV‐FFF beam) for single‐isocenter/multi‐lesions VMAT lung SBRT with curative therapeutic biological effective dose to each lesion (>100 Gy) has been presented. Utilizing PO algorithm during VMAT lung SBRT plan optimization potentially reduces MLC complexity and beam‐on time while providing similar target coverage and similar dose to the OAR. PO MLC algorithm was shown to be advantageous for treating multiple dispersed lung lesions as described here. Therefore, to minimize MLC complexity and consequently beam‐on time we strongly recommend utilizing PO algorithm (if available) for multi‐lesion VMAT SBRT plan optimization, thereby reducing the total MU, MLC leakage and transmission and potentially minimizing unwanted dose to the patients.

## 5 CONCLUSIONS

With PO algorithm, larger MLC openings and less total MU led to shorter beam‐on time (maximum reduction up to 2.0 min) while synchronously treating multiple lung lesions using a single‐isocenter VMAT SBRT plan. For multiple synchronous lesions, PO MLC algorithm improved treatment efficiency without compromising plan quality when compared to the PRO algorithm. Faster treatment time can potentially reduce intrafraction motion errors and improve patient compliance, especially in elderly patients. Our preliminary data suggest that PO MLC algorithm could be useful for future clinical VMAT lung SBRT plan optimization.

## CONFLICT OF INTEREST

The authors declare no conflict of interest.

## References

[1] Timmerman R , Paulus R , Galvin J , et al. Stereotactic body radiation therapy for inoperable early stage lung cancer. JAMA. 2010;303:1070–1076. 20233825 10.1001/jama.2010.261 PMC2907644

[2] Benedict S , Yenice K , Followill D , et al. Stereotactic body radiation therapy: the report of AAPM Task Group 101. Med Phys. 2010;37:4078–4101. 20879569 10.1118/1.3438081

[3] A Randomized Phase II Study Comparing 2 Stereotactic Body Radiation Therapy (SBRT) Schedules For Medically Inoperable Patients with Stage I Peripherial Non‐Small Cell Lung Cancer; RTOG 0915; 2014:1–67.

[4] Onishi H , Shirato H , Nagata Y , et al. Stereotactic body radiotherapy (SBRT) for operable stage I non‐small‐cell lung cancer: Can SBRT be comparable to surgery? Int J Radiat Oncol Biol Phys. 2011;81:1352–1358. 20638194 10.1016/j.ijrobp.2009.07.1751

[5] Sandhu A , Lau S , Rahn D , et al. Stereotactic body radiation therapy in octogenarians with stage I lung cancer. Clin Lung Cancer. 2014;15:131–135. 24157245 10.1016/j.cllc.2013.08.007

[6] Al‐Hallaq H , Chmura S , Salama J , et al. Rational of technical requirements for NRG‐BR001: the first NCI‐sponsored trial of SBRT for the treatment of multiple metastases. Pract Radiat Oncol. 2016;6:e291–e298. 27345129 10.1016/j.prro.2016.05.004 PMC5099083

[7] Zhang Y , Chen Y , Qiu J , et al. Dosimetric comparisons of lung SBRT with multiple metastases by two advanced planning systems. Int J Med Phys Clin Eng Radiat Oncol. 2014;3:252–261.

[8] Li Q , Mu J , Gu W , et al. Frameless stereotactic body radiation therapy for multiple lung metastases. J Appl Clin Med Phys. 2014;15:105–115. 10.1120/jacmp.v15i4.4737 PMC5875519 25207400

[9] Quan K , Xu K , Lalonde R , et al. Treatment plan technique and quality for single‐isocenter stereotactic ablative radiotherapy of multiple lung lesions with volumetric‐modulated arc therapy or intensity‐modulated radiosurgery. Front Oncol. 2015;5:1–9. 26500888 10.3389/fonc.2015.00213 PMC4594030

[10] Navarria P , Ascolese AM , Mancosu P , et al. Volumetric modulated arc therapy with flattening filter free (FFF) beams for stereotactic body radiation therapy (SBRT) in patients with medically inoper‐able early stage non‐small cell lung cancer (NSCLC). Radiother Oncol. 2013;107:414. 23725859 10.1016/j.radonc.2013.04.016

[11] Sanford L , Molloy J , Kumar S , et al. Evaluation of plan quality and treatment efficiency for single‐isocenter/two‐lesion lung stereotactic body radiation therapy. J Appl Clin Med Phys. 2019;20:117–127. 10.1002/acm2.12500 PMC6333146 30548205

[12] Trager M , Salama J , Yin F‐F , Adamson J . SBRT treatment of multiple extracranial oligometastases using a single isocenter with distinct optimizations. J Radiosurg SBRT. 2017;4:365–273. PMC5658822 29296451

[13] Pokhrel D , Sanford L , Halfman M , Molloy J . Potential reduction of lung dose via VMAT with jaw tracking in the treatment of single‐isocenter/two‐lesion lung SBRT. J Appl Clin Med Phys. 2019;21:1–9. 10.1002/acm2.12580 PMC6523009 30955251

[14] Eclipse Photon and Electron Algorithms 13.7 Reference Guide, 2018.

[15] Shende R , Gupta G , Patel G , Kumar S . Assessment and performance evaluation of photon optimizer (PO) vs. dose volume optimizer (DVO) for IMRT and progressive resolution optimizer (PRO) for RapidArc planning using a virtual phantom. Int J Cancer Ther Oncol. 2016;4:437.

[16] Binny D , Kairn T , Lancaster C , et al. Photon optimizer (PO) vs progressive resolution optimizer (PRO): a conformality‐ and complexity‐based comparison for intensity‐modulated arc therapy plans. Med Dosim. 2017;43:267–275. 29079336 10.1016/j.meddos.2017.10.003

[17] Jiang F , Wu H , Yue H , et al. Photon optimizer (PO) prevails over progressive resolution optimizer (PRO) for VMAT planning with or without knowledge‐based solution. J Appl Clin Med Phys. 2017;18:9–14. 10.1002/acm2.12038 PMC5689948 28300375

[18] Liu H , Sintay B , Pearman K , et al. Comparison of the progressive resolution optimizer and photon optimizer in VMAT optimization for stereotactic treatments. J Appl Clin Med Phys. 2018;19:155–162. 29781138 10.1002/acm2.12355 PMC6036352

[19] Kry SF , Bednarz B , Howell RM , et al. AAPM TG 158: measurement and calculation of doses outside the treated volume from external‐beam radiation therapy. Med Phys. 2017;44:e391–e492. 28688159 10.1002/mp.12462

[20] Baker R , Han G , Sarangkasiri S , et al. Clinical and dosimetric predictors of radiation pneumonitis in a large series of patients treated with stereotactic body radiation therapy to the lung. Int J Radiat Oncol Biol Phys. 2013;85:190–195. 22929858 10.1016/j.ijrobp.2012.03.041

[21] Vanetti E , Nicolini G , Nord J , et al. On the role of the optimization algorithm of RapidArc((R)) volumetric modulated arc therapy on plan quality and efficiency. Med Phys. 2011;38:5844. 22047348 10.1118/1.3641866

